# The eggshell is required for meiotic fidelity, polar-body extrusion and polarization of the *C. elegans *embryo

**DOI:** 10.1186/1741-7007-4-35

**Published:** 2006-10-16

**Authors:** Wendy L Johnston, Aldis Krizus, James W Dennis

**Affiliations:** 1 Samuel Lunenfeld Research Institute, Mount Sinai Hospital, 600 University Ave. Toronto, ON, M5G 1X5, Canada; 2 Department of Medical Genetics & Microbiology, University of Toronto, ON, Canada

## Abstract

**Background:**

Fertilization restores the diploid state and begins the process by which the single-cell oocyte is converted into a polarized, multicellular organism. In the nematode, *Caenorhabditis elegans*, two of the earliest events following fertilization are secretion of the chitinous eggshell and completion of meiosis, and in this report we demonstrate that the eggshell is essential for multiple developmental events at the one-cell stage.

**Results:**

We show that the GLD (Germline differentiation abnormal)-1-regulated hexosamine pathway enzyme, glucosamine-6-phosphate N-acetyltransferase (GNA)-2, is required for synthesis of uridine diphosphate-N-acetylglucosamine (UDP-GlcNAc), the substrate for eggshell chitin synthesis by chitin synthase-1 (CHS-1). Furthermore, while *chs-1(RNAi) *or combined RNAi with the chitin-binding proteins, CEJ-1 and B0280.5, does not interfere with normal meiotic timing, lagging chromosomes are observed at meiosis, and polar-body extrusion fails. We also demonstrate that chitin, and either CEJ-1 or B0280.5, are essential for the osmotic/permeability barrier and for movement of the sperm pronucleus/centrosome complex to the cortex, which is associated with the initiation of polarization.

**Conclusion:**

Our results indicate that the eggshell is required in single-cell *C. elegans *development, playing an essential role in multiple actin-dependent early events. Furthermore, the earliest meiotic roles precede osmotic barrier formation, indicating that the role of the eggshell is not limited to generation of the osmotic barrier.

## Background

During oocyte development, inhibition of mitogen-activated protein kinase (MAPK) signaling causes arrest at meiotic prophase I. Subsequent maturation, which results in breakdown of the germinal vesicle (nuclear envelope) and assembly of the meiotic spindle, requires relief of MAPK inhibition. In *Caenorhabditis elegans*, this relief is provided by major sperm protein (MSP), budded off from sperm stored in the spermatheca [1,2]. MSP displaces ephrin bound to oocyte VAB-1 receptors, resulting in MAPK activation and oocyte maturation. MSP also binds non-VAB receptors on oocyte and gonadal sheath cell membranes, inducing ovulation of the mature oocyte into the spermatheca, the site of fertilization. Fertilization activates the anaphase-promoting complex/cyclosome (APC/C), triggering progression past anaphase I [3]. Concomitantly, fertilization signals the rapid assembly of a chitinous eggshell that surrounds the developing embryo until hatching.

The nematode eggshell can have up to five layers, although in most species, including *C. elegans*, it is a trilamellate structure, comprised of an outer vitelline layer, a middle chitin-containing layer and an inner lipid-rich layer [4-7]. Detailed ultrastructural studies of the *C. elegans *eggshell are lacking. However, electron micrographic studies of *Ascaris lumbricoides *[8] revealed that shortly after sperm penetration, the outer plasma membrane-like layer separates from the egg cytoplasm, resulting in a dense outer vitelline layer. Underlying the vitelline layer is a structureless zone that subsequently becomes filled with chitin and protein, resulting in the formation of the mechanically resistant middle layer of the shell. Specific proteins in this middle layer have not been identified, but proteins with chitin-binding domains are likely candidates. In *C. elegans*, these include T10E10.4, F23F12.8, M03E7.4, R02F2.4, K04H4.2A, C39D10.7, W03F11.1, W02A2.3, H02I12.1, B0280.5 and CEJ-1, proteins predicted to have Peritrophin-A domains, a conserved chitin-binding domain found in peritrophic matrix proteins of insects and in animal chitinases [9,10]. Two of these proteins, CEJ-1 and B0280.5, have recently been shown to bind chitin and to be modified by chondroitin addition [11]. Moreover, chondroitin deficiency in squashed vulva (Sqv) mutants results in embryos in which the space between the eggshell and the embryo is missing, and in which cytokinesis at the one-cell stage is defective [12]. These results suggest that CEJ-1 and B0280.5 are likely to play an important role as components of the eggshell.

Coincident with the deposition of chitin and protein into the middle layer of the shell, the inner proteolipid layer of the eggshell (the proposed permeability barrier) is formed by extrusion of embryonic cytoplasmic refringent granules, and the first polar body is extruded into this layer. By the time of pseudocleavage in the one-cell embryo, the trilamellate eggshell is separated from the embryo plasma membrane by a clear zone, which may be the precursor of the perivitelline fluid (PVF) that surrounds later stage embryos. In 3-day-old *Ascaris suum *embryos, the PVF has been shown to contain a number of proteins, including the fatty acid-binding protein, As-p18, which has been suggested to play a role in maintaining the barrier function of the inner eggshell layer [13].

**Figure 1:**
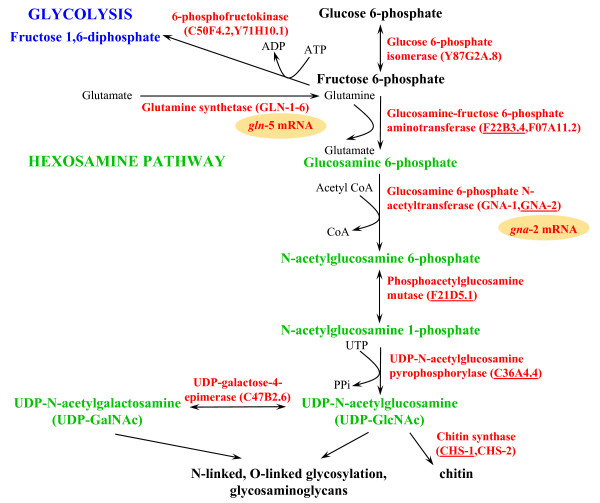
**Hexosamine pathway for biosynthesis of UDP-GlcNAc and UDP-GalNAc**. Fructose 6-phosphate enters glycolysis by conversion to fructose 1,6-diphosphate, catalyzed by 6-phosphofructokinase, or enters the hexosamine pathway by conversion to glucosamine 6-phosphate, catalyzed by glucosamine-fructose 6-phosphate aminotransferase (GFAT). Two enzymes required for UDP-GlcNAc synthesis (glutamine synthetase-5 (GLN-5)) and glucosamine 6-phosphate N-acetyltransferase-2 (GNA-2)) are targets of GLD-1 translational repression in *C. elegans *(shown by a gold oval encompassing the mRNA). *C. elegans *homologues are in brackets; those underlined are oogenesis enriched [56] and have an osmotically-sensitive embryonic lethal phenotype by RNAi [20]; this study.

Chitin ([(GlcNAcβ1-4GlcNAc)_n_]) is polymerized from the sugar nucleotide donor, uridine diphosphate-N-acetylglucosamine (UDP-GlcNAc), synthesized by the hexosamine pathway (Figure 1) [14]. Eggshell chitin production places a sudden and high demand on this pathway, with as much as 50% of embryonic glycogen proposed to be required for UDP-GlcNAc synthesis in *Ascaris megalocephala *[15]. The *C. elegans *eggshell can be removed at the two-cell stage, without interrrupting development, at least until gastrulation [16]. This has led to the suggestion that the function of the chitinous eggshell is restricted to mechanical support of the developing embryo from gastrulation onwards. However, an earlier embryonic lethal phenotype results from RNA interference (RNAi), with enzymes catalyzing any one of the five hexosamine pathway steps leading to chitin synthesis (Figure 1) [17-20]. Furthermore, transcripts for two key enzymes in this pathway, glucosamine-6-phosphate N-acetyltransferase (GNA)-2 and glutamine synthetase (GLN)-5, are tightly regulated by the germline translational repressor, GLD-1 (Figure 1) [21,22]. Taken together, these results suggest that the chitinous eggshell is developmentally essential before the two-cell stage.

Many of the developmental events that follow fertilization require asymmetric or focal actomyosin contraction, which may depend on eggshell assembly. For example, polar-body cytokinesis is highly asymmetric and depends on non-muscle myosin (NMY)-2 enriched at the contractile ring [23,24]. Polar-body extrusion also requires the actin/myosin cross-linking protein, ANI-1, the myosin light chain homologue, MLC-4, and the microfilament organizing proteins, profilin (PFN)-1 and CYK (Cytokinesis defect)-1 [23-25]. Following polar-body extrusion, the embryonic cortex undergoes a period of membrane ruffling that is dependent on focal enrichment of the actomyosin cytoskeleton at the base of the ruffles [26]. Ruffling requires ANI-1, NMY-2, PFN-1 and CYK-1, and is coincident with cytoplasmic flow and the movement of the sperm pronucleus/centrosome complex (SPCC) towards the cortex [27]. SPCC-cortical association marks the posterior of the embryo [28], and is associated with the initiation of anterior-posterior (A-P) polarization, which culminates in an asymmetric first cell division, generating two daughter cells (AB and P_1_) with distinct developmental potentials [29,30]. Coincident with SPCC-cortical association is the local clearing of NMY-2 foci and local relaxation of actomyosin contraction. The resultant non-contractile cortex expands anteriorly to the approximate halfway point, terminating in the pseudocleavage furrow, which demarcates the smooth PAR (Partitioning abnormal)-2/PAR-1-binding posterior cortex from the contractile PAR-3/PAR-6/PKC-3-binding anterior cortex [26,30]. This highly asymmetric contraction and P-A cortical flow is associated with an opposing A-P cytoplasmic flow, which transports cytoplasmic determinants, including P granules, to the posterior [30,31].

Cortical and cytoplasmic flows at polarization are dependent on PAR-2 (ring finger), PAR-3 (PDZ-containing), PAR-4 (Ser/Thr kinase), PAR-6 (PDZ-containing), and MEX-5 and MEX-6, two related cytoplasmic zinc-finger proteins [29]. PAR-1 (Ser/Thr kinase) appears dispensable for flows, but is required later to negatively regulate the activity and stability of MEX-5/MEX-6, and to restrict P granules and PIE-1 to the posterior [31]. PAR-1, -2, -3 and -6 are asymmetrically distributed, and PAR-3/PAR-6 polarization requires CDC-42, a member of the Rho family of guanosine triphosphatases (GTPases) that, in mammalian axons, causes actin reorganization and elongation [32-34]. In addition to polarizing the cortex and the cytoplasm, *C. elegans *PARs are required for the unequal pulling forces on the anterior and posterior mitotic spindle poles that result in an asymmetric first cell division [35-37]. Polarization and asymmetric division also require the DYRK-related minibrain kinase-2 (MBK-2). MBK-2 stimulates the CUL-3/MEL-26 E3 ubiquitin ligase-dependent destruction of MEI-1 and MEI-2, two katanin-related microtubule-severing proteins. MEI-1 and MEI-2 maintain the smaller meiotic spindle, and their destruction is required to permit polymerization of the larger mitotic spindle [38-44]. MBK-2 also targets the oocyte maturation factor, OMA-1, for CUL-2/ZYG-11 E3 ubiquitin ligase-dependent destruction. This relieves the OMA-1 block to CUL-2/ZIF-1 E3 ubiquitin ligase-dependent destruction of PIE-1, which contributes to the destruction of PIE-1 in the anterior of the embryo [45].

Progression past meiosis metaphase I requires activation of the APC/C. Complete loss of function of genes encoding APC subunits results in Mat (metaphase to anaphase transition-defective) embryos with either no polarization or reversed polarization [28]. Partial loss of function of APC can support the completion of meiosis, but results in embryos with combined polarity and osmotic-sensitivity defects (Pod phenotype of *mat 1–3*, *emb-27, emb-30*) [46,47]. The Pod class is diverse, including not only APC subunit genes, but also genes encoding a coronin-like actin-binding protein (POD-1), two genes encoding enzymes required for fatty-acid biosynthesis [acetylCoA carboxylase (POD-2) and NADPH-dependent cytochrome P450 reductase (EMB-8)] and a gene encoding the Rho family GTPase, CDC-42 [5,32,48-50]. In this study, we identify *gna-2(qa705) *as a novel Pod mutant, and demonstrate that eggshell chitin and two functionally redundant chitin-binding proteins, CEJ-1 and B0280.5, are essential for error-free segregation at meiosis, for extrusion of the polar bodies and for SPCC association with the cortex. These results form the basis of a model of *C. elegans *embryonic development that includes an essential role for the eggshell in actomyosin-dependent events at the one-cell stage.

## Results

### *gna-2(qa705) *is UDP-GlcNAc-deficient, and genetic rescue results in a Him phenotype

**Figure 2:**
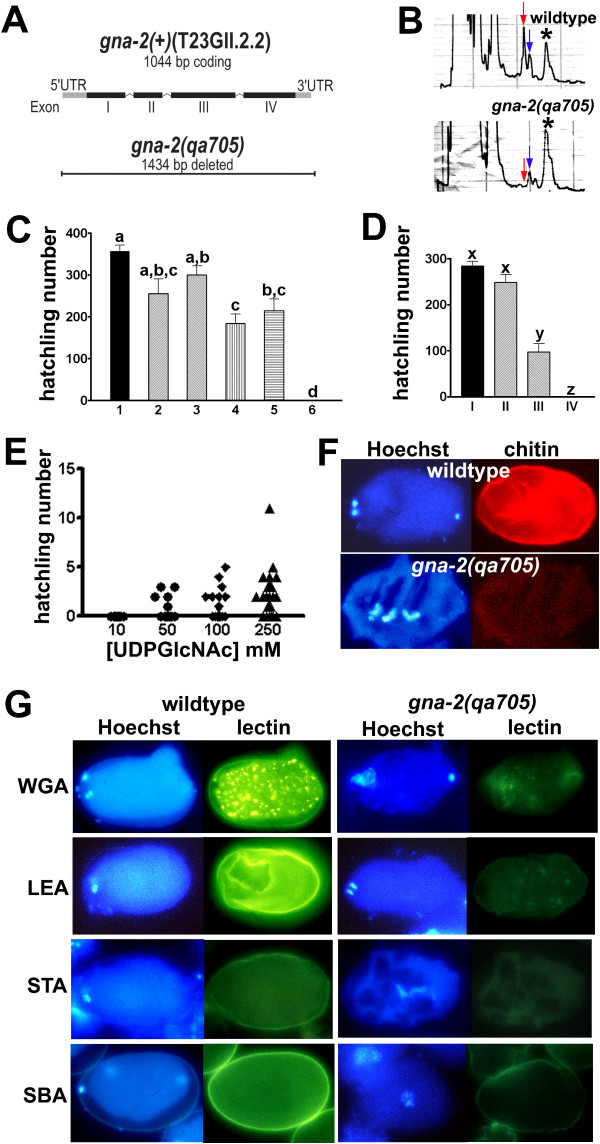
***gna-2(qa705) *is deficient in UDP-GlcNAc and its products**. (A) *gna-2(qa705) *has a 1434-bp deletion that removes the entire coding sequence of *gna-2 (T23G11.2.2)*. (B) HPLC traces (A_254 _nm) showing UDP-GlcNAc (red arrow), UDP-GalNAc (blue arrow) and UDP-Glc + UDP-Gal (asterisk) in *gna-2(qa705) *or wild type. (C) Hatchling number of (1) wild type, (2) *sup-46(qa707*), (3) *sup-46(qa708)*, (4) *gna-2(qa705)sup-46(qa707)*, (5) *gna-2(qa705)sup-46(qa708) *or (6) *gna-2(qa705)*. Genotypes with different letters are significantly different (*p *< 0.05, *n *= 9, mean ± SEM). (D) Hatchling number of (I) wild type, (II) *gna-1(RNAi)*, (III) *gna-2(qa705)sup-46(qa707) *or (IV) *gna-2(qa705)sup-46(qa707)*; *gna-1(RNAi)*. Genotypes with different letters are significantly different (*p *< 0.05, *n *= 10, bars are mean ± SEM). (E) Hatchling number of *gna-2(qa705) *following injection of UDP-GlcNAc into one distal gonad arm. Each point represents hatchlings of one injected hermaphrodite. (F) Wild type or *gna-2(qa705) *stained for DNA (Hoechst) and chitin or (G) DNA (Hoechst) and epitopes for the following lectins: WGA, LEA, STA, SBA. (F,G) Embryos are at the one-cell stage. Wild-type embryos are oriented with anterior (maternal DNA, polar-body end) on the left. *gna-2(qa705) *does not extrude polar bodies but where maternal meiotic DNA was visible, it was oriented on the left.

The *C. elegans *genome has two *gna *homologues, *gna-1 *and *gna-2*. RNAi with *gna-1 *results in a wild-type phenotype, while RNAi with *gna-2 *is embryonic lethal (). To explore the role of GNA-2 in *C. elegans *development, we isolated (*qa705*), an allele lacking the entire coding sequence (Figure 2A).

*gna-2(qa705) *males are fertile, and hermaphrodites are viable but 100% penetrant for the maternal effect embryonic lethal (Mel) phenotype. An extrachromosomal array containing the coding sequence for *gna-2 *(nucleotides 20340–23478 in reference cosmid T23G11) partially rescued the *gna-2(qa705) *embryonic lethal phenotype and resulted in a high incidence of males (Him) phenotype. Hatchling numbers/hermaphrodite, % Him and number (*n*) of hermaphrodites assayed were as follows. (i) Wild type with no extrachromosomal array: 261 hatchlings, 0.2% Him, *n *= 10; (ii) wild type carrying an extrachromosomal array of the visible Rol6 marker, pRF4: 219 hatchlings, 0.1% Him, n = 10; and (iii) two independent lines of *gna-2(qa705) *carrying the visible Rol6 marker, pRF4, and also carrying an extrachromosomal array bearing a translational fusion of *gna-2::GFP *(line 1: 48 hatchlings, 2.7% Him, *n *= 10; line 2: 39 hatchlings, 4.6% Him, *n *= 10). In adult *gna-2(qa705) *hermaphrodites, UDP-GlcNAc was undetectable (Figure 2B, red arrow), and its epimer, UDP-GalNAc, was decreased to 19% of wild-type levels (Figure 2B, blue arrow). Three alleles *(qa707, qa708, qa709*) of a single suppressor *(sup-46) *of *gna-2(qa705) *were identified, and *sup-46 *required *gna-1 *for suppression of lethality, suggesting that UDP-GlcNAc synthesis is essential for embryonic development (Figure 2C,D). Furthermore, the *gna-2(qa705) *Mel phenotype was partially rescued by injection of 50–250 mM UDP-GlcNAc into the distal germline (Figure 2E). These results demonstrate that the Mel phenotype of *gna-2(qa705) *results from loss of GNA-2 catalytic activity, which causes UDP-GlcNAc deficiency in the germline or early embryo. Thus, *de novo *synthesis of UDP-GlcNAc is essential for early development in *C. elegans*. The UDP-GlcNAc end product, chitin, was markedly reduced in embryos dissected from *gna-2(qa705) *hermaphrodites (henceforth referred to as *gna-2(qa705) *embryos) (Figure 2F), as was reactivity with the lectins WGA, LEA, STA and SBA (Figure 2G). In wild-type embryos, WGA staining revealed transient cortical-membrane punctae that appeared by the first meiotic division and disappeared by the two-cell stage (Figure 2G). LEA, STA, SBA and CBD-F staining were not punctate, indicating that WGA reactivity in wild-type embryos reveals a non-chitin glycoconjugate(s) (WGA-G). WGA binds with high affinity to α2–3-linked sialic acids in mammalian cells. However, these structures are absent in *C. elegans*, and Natsuka et al [51] have shown that WGA binding in *C. elegans *most likely reflects α-GalNAc modification of mucin-type glycoproteins.

### Meiosis and polar-body extrusion are defective in *gna-2(qa705)*

**Figure 3:**
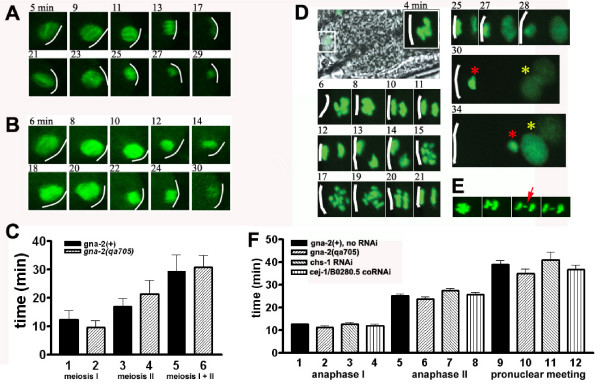
***gna-2(qa705) *has normal meiotic timing, but fails to extrude polar bodies and has 12 sister chromatids at meiosis II**. *In utero *recording of (A) wild-type embryo expressing tubulin::GFP, showing first meiotic spindle (top row) and second meiotic spindle (bottom row) or (B) *gna-2(qa705) *embryo expressing tubulin::GFP, showing first meiotic spindle (top row) and second meiotic spindle (bottom row). (B,C) Times are from entry into the spermatheca. (C) Time from entry into spermatheca until waning of first meiotic spindle (bars 1,2), from waning of first meiotic spindle until waning of second meiotic spindle (bars 3,4) or total time from entry into spermatheca until waning of second meiotic spindle (bars 5,6), in wild-type embryos expressing tubulin::GFP, (n = 3) or *gna-2(qa705) *embryos expressing tubulin::GFP, (*n *= 4). Bars are mean ± SEM. For each comparison, *gna-2(qa705) *was not significantly different from wild type (*p *> 0.05). (D) *gna-2(qa705) *expressing histone H2B::GFP, showing meiotic DNA segregation. Times are from entry into the spermatheca. Large, black and white image (4-min time point) shows the embryo *in utero*, with the box indicating the maternal DNA shown enlarged (GFP signal) in the upper right hand corner. Unextruded second polar body is indicated by a red asterisk at the 30 and 34 min time points, and maternal and paternal pronuclei, which have met, are indicated by a yellow asterisk at the 30- and 34-min time points. (A,B,D) White line indicates embryonic cortex. (E) *gna-2(qa705*) expressing histone H2B::GFP, showing stray chromatin (red arrow) (distinct from two major masses segregated at meiotic anaphase II). (F) Time from entry into spermatheca until meiotic anaphase I (bars 1–4), until meiotic anaphase II (bars 5–8) or until pronuclear meeting (bars 9–12), in wild type (*n *= 11), *gna-2(qa705) *(*n *= 13), *chs-1(RNAi) *(*n *= 9) or *cej-1+B0280.5(coRNAi) *(*n *= 14), expressing histone H2B::GFP. Bars are mean ± SEM. For each comparison (wild type, *gna-2(qa705), chs-1(RNAi) *and *cej-1+B0280.5(coRNAi)*) there were no significant differences (*p *> 0.05).

Live *in utero *recordings of *gna-2(qa705) *embryos expressing tubulin::GFP showed meiotic spindles that wax and wane with normal timing (Figure 3A,C). Meiosis I+II was almost identical for wild type (29 min) and *gna-2(qa705) *(31 min). Furthermore, the time during which a meiosis II spindle could be visualized in wild type (8 min) and *gna-2(qa705) *(12 min) (Figure 3A,B; bottom rows) is consistent with the timing for wild type reported by Liu et al[53], (13 min), but very different from the timing for *cul-2(RNAi) *(meiosis II spindle still present at 36 min), which has a perdurant meiosis II spindle.

As an independent measure of meiotic timing, we examined embryos expressing histone H2B::GFP (Figure 3D,F). Results for wild-type meiotic timing (13 min for meiosis I, 25 min for meiosis II) were similar to those previously reported for wild type (15–17 min for meiosis I, 30–31 min for meiosis II) [52,53]. Furthermore, *gna-2(qa705) *had meiosis I and meiosis II timings similar to wild type. This is very different from results reported for *cul-2(RNAi) *or *zyg-11(RNAi)*, in which meiosis I timing was normal, but meiosis II time was extended (60–75 min) [52,53]. Combined with the tubulin::GFP results, the histone H2B::GFP results support the conclusion that *gna-2(qa705) *does not have a perdurant meiosis II spindle. Using the histone H2B::GFP, we detected stray chromatin (distinct from the two masses segregated at anaphase) in 8% of *gna-2(qa705) *embryos at anaphase I, and 15% of *gna-2(qa705) *embryos at anaphase II, suggesting chromosome nondisjunction (Figure 3E, red arrow, Table 1). Additionally, a polar body was not extruded following anaphase I, and all maternal DNA entered meiosis II, resulting in 12 sister chromatid pairs at prophase II (100% of embryos), rather than the normal number of 6 (Figure 3D, 17 min time point, Table 1). A second polar body formed after anaphase II, but was not extruded (100% of embryos; Table 1). Whereas polar-body extrusion was defective, maternal and paternal pronuclei decondensed and met with normal timing (Figure 3D, yellow asterisk, Figure 3F). The unextruded second polar body (Figure 3D, red asterisk) often migrated to meet the pronuclei, although its migration usually lagged behind that of the maternal pronucleus.

**Table 1:** Summary of meiotic phenotype in *gna-2(qa705)*, *chs-1(RNAi) *or *cej-1+B0280.5(coRNAi)*^a^

	**control^b^**	***gna-2(qa705)*^c^**	***chs-1(RNAi)*^d^**	***cej-1+B0280.5(coRNAi)*^e^**
**1^st ^polar body not extruded, 12 sister chromatids at meiosis II**	0	100	100	76
**2^nd ^polar body not extuded**	0	100	100	82
**stray chromatin at meiosis I^f^**	0	8	0	6
**stray chromatin at meiosis II^f^**	0	15	10	12
**SPCC failed to contact cortex^g^**	0	69	30	47
**no pseudocleavage furrow**	0	100	100	94

Numbers are % of embryos displaying phenotype

^**a **^embryos examined *in utero *using a Leica DMLSFA Confocal Microscope.

^**b **^wild type expressing histone H2B::GFP; n = 10

^**c **^*gna-2(qa705) *expressing histone H2B::GFP; n = 13

^**d **^*chs-1(RNAi) *expressing histone H2B::GFP; n = 10

^**e **^*cej-1*+*B0280.5(coRNAi) *expressing histone H2B::GFP; n = 17

^**f **^3rd, smaller mass of chromatin, separate from 2 major masses of chromatin segregated at anaphase

^**g **^sperm pronucleus failed to come within 1/2 sperm pronucleus diameter of the cortex

### *gna-2(qa705) *is a novel Pod gene

**Figure 4:**
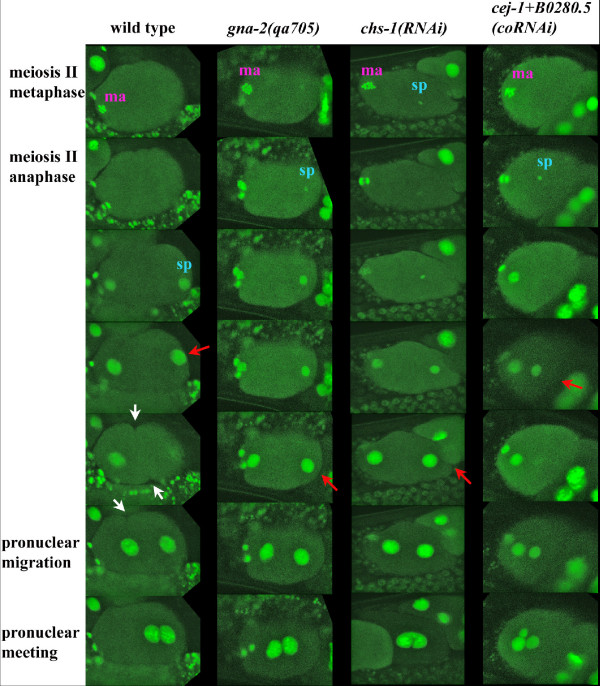
**Failure of SPCC-cortical association and pseudocleavage furrow formation in *gna-2(qa705)*, *chs-1(RNAi) *and *cej-1+B0280.5(coRNAi)***. Columns of images are a developmental series of individual wild-type, *gna-2(qa705)*, *chs-1(RNAi) *or *cej-1+B0280.5(coRNAi) *embryos, from meiosis II metaphase to pronuclear meeting. In wild type, the sperm pronucleus (sp) touches the cortex (indicated by a red arrow) and a pseudocleavage furrow can be seen (indicated by white arrows) before and during pronuclear migration. In *gna-2(qa705)*, *chs-1(RNAi) *or *cej-1+B0280.5(coRNAi) *a sperm pronucleus (sp) decondenses but fails to associate closely with the cortex (indicated by a red arrow), and a pseudocleavage furrow was not seen. Maternal (ma) DNA (11–12 sister chromatid pairs can be seen at meiosis II metaphase in *gna-2(qa705) *and *chs-1(RNAi)*). Images are of live embryos expressing histone H2B::GFP, recorded *in utero*.

**Figure 5:**
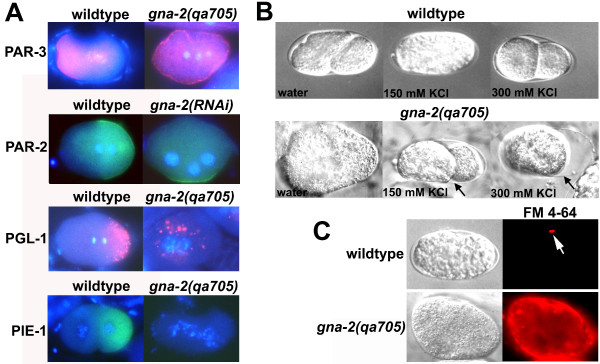
**GNA-2 deficiency results in a polarity-osmotic defective (Pod) phenotype**. (A) Wild type or *gna-2(qa705) *stained for DNA (Hoechst stain; blue) and PAR-3 (pink), PGL-1 (pink) or PIE-1 (green), or wild type expressing PAR-2::GFP fed OP50 (control) or *gna-2 *RNAi and stained for DNA (Hoechst; blue) and GFP (green) (second row). Embryos are at the one-cell stage except wild type stained for PIE-1, which is at the two-cell stage. Wild-type embryos are oriented with anterior (maternal DNA, polar-body end) on the left. *gna-2(qa705) *or *gna-2(RNAi) *does not extrude polar bodies therefore it was not possible to determine the maternal end. Images are overlays of Hoechst and antibody staining. (B) DIC images showing osmotic sensitivity of wild type or *gna-2(qa705) *dissected in water, 150 mM KCl or 300 mM KCl. Arrows point to the eggshell. (C) Plasma-membrane staining of *gna-2(qa705) *with the lipophilic dye, FM 4–64 (red). In wild type, only the first polar body stains (white arrow). DIC images are shown on the left.

Live *in utero *recording also showed that the SPCC failed to associate closely with the cortex in *gna-2(qa705) *(69% of embryos) (Table 1, Figure 4). In wild-type embryos, SPCC-cortical association correlates with the initiation of polarization, which results in the anterior spread of a PAR-1,2-binding smooth posterior cortex that culminates in the pseudocleavage furrow. In addition to showing failed SPCC-cortical association, a pseudocleavage furrow was never observed in *gna-2(qa705) *(100% of embryos; Table 1, Figure 4). Furthermore, cortical polarity was defective in *gna-2(qa705) *embryos, as indicated by a failure to segregate PAR-3 to the anterior cortex (Figure 5A), and cortical PAR-2 was reduced and mislocalized following GNA-2 depletion by *gna-2 *RNAi (Figure 5A, Table 2; 150 mM KCl treatment). Cytoplasmic polarity was also defective, as posterior localization of P granules was not observed and PIE-1 was undetectable (Figure 5A).

**Table 2:** PAR-2::GFP polarization following osmotic support in EGM (30 min)^a^

	**150 mM KCl^b,c^**			**EGM (30 min)^d,e^**		
	no asymmetry^**f**^	incorrect/incomplete asymmetry^**g**^	correct asymmetry^**h**^	no asymmetry^**f**^	incorrect/incomplete asymmetry^**g**^	correct asymmetry^**h**^

**control^i^**	0	0	100	0	0	100
***gna-2(RNAi)***	100	0	0	40	50	10
***chs-1(RNAi)***	15	85	0	70	20	10
***cej-1+B0280.5(coRNAi)***	25	63	13	50	50	0

^**a **^PAR-2::GFP was examined in single-cell embryos in which 2 discrete Hoechst-staining structures were in close proximity to one another in the cytoplasm. *gna-2(RNAi), chs-1(RNAi) *and *cej-1+B0280.5(coRNAi) *fail to extrude the 2nd polar body; therefore, a 3rd Hoechst-staining structure could often be discerned. Numbers are % of embryos displaying phenotype

^**b **^*gna-2(RNAi), chs-1(RNAi) *and *cej-1+B0280.5(coRNAi) *embryos are osmotically sensitive and burst without osmotic support; therefore, embryos were dissected as quickly as possible in 150 mM KCl (<5 min) and frozen immediately

^**c **^control, n = 10; *gna-2(RNAi)*, n = 8; *chs-1(RNAi)*, n = 6; *cej-1+B0280.5(coRNAi)*, n = 24

^**d **^embryos were dissected in minimal EGM and incubated at 22°C for 30 min

^**e **^control, n = 10; *gna-2(RNAi)*, n = 10; *chs-1(RNAi)*, n = 10; *cej-1+B0280.5(coRNAi)*, n = 8

^**f **^PAR-2::GFP undetectable or uniform cortical

^**g **^PAR-2::GFP lateral or incompletely anterior/posterior. Since loss of function of *gna-2*, *chs-1 *or *cej-1+B0280.5 *prevents polar body extrusion, it was not possible to definitively identify anterior versus posterior

^**h **^PAR-2::GFP posterior

^**i **^wild type fed OP50

Differential interference contrast (DIC) imaging (Leica Leitz DMRB) revealed that *gna-2(qa705) *embryos had some form of eggshell (Figure 5B, arrows); however, the shell was deficient in chitin and other lectin-reactive glycoconjugates (Figure 2F,G) and did not maintain a rigid oval shape in the uterus. *gna-2(qa705) *embryos were also osmotically sensitive, swelling in H_2_O and shrinking in 300 mM KCl, and were permeable to the lipophilic dye, FM 4–64, consistent with an osmotic/permeability barrier defect (Figure 5B,C). Together with the polarity defects, this identifies *gna-2(qa705) *as a novel Pod mutant. It is possible that polarization defects in Pod mutants might be secondary to osmotic defects, as has been shown for the cytokinesis defect of *cyk-3 *mutants [54]. As such, the chitinous eggshell may be essential to provide an osmotically appropriate environment for A-P polarization. To determine if GNA-2-deficient embryos could polarize if provided with synthetic osmotic support, worms expressing PAR-2::GFP were subjected to *gna-2 *RNAi, and embryos were dissected and incubated in minimal embryonic growth medium (EGM) [55]. EGM provided little rescue of polarization (Table 2), suggesting that polarization defects in GNA-2-deficient embryos may be dissociable from osmotic sensitivity. However, it is also possible that EGM did not provide adequate and/or timely osmotic support.

### RNAi with chitin synthase-1 phenocopies *gna-2(qa705)*

**Figure 6:**
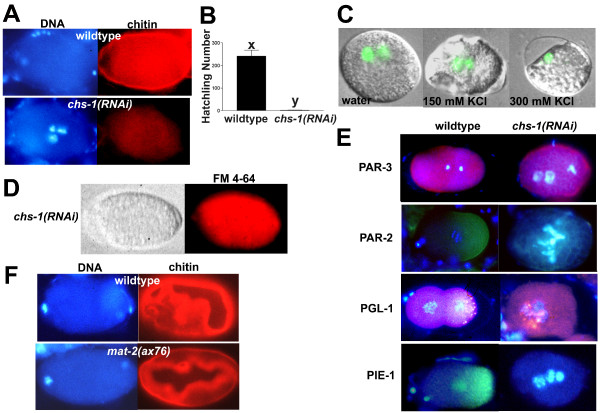
***chs-1(RNAi) *results in a *gna-2(qa705)*-like Pod phenotype**. (A) DNA (Hoechst) and chitin staining of wild type or *chs-1(RNAi)*. (B) Hatchling number of wild type or *chs-1(RNAi) *(mean ± SEM, *n *= 11, x, y: significantly different, *p *< 0.05). (C) Osmotic sensitivity of *chs-1(RNAi) *embryos expressing histone H2B::GFP, dissected in water, 150 mM KCl or 300 mM KCl. Images are overlays of histone H2B::GFP (chromatin; green) and DIC. (D) FM 4–64 permeability of *chs-1(RNAi)*. DIC image is on the left. (E) Wild type or *chs-1(RNAi) *stained for DNA (Hoechst; blue) and PAR-3 (red), PGL-1 (pink) or PIE-1 (green), or wild type expressing PAR-2::GFP fed OP50 (control) or *chs-1 *RNAi and stained for DNA (Hoechst; blue) and GFP (green) (second row). Images are overlays of Hoechst and antibody staining. (F) DNA (Hoechst) and chitin staining of wild type or *mat-2(ax76)*. (A,E,F) Embryos are at the one-cell stage except wild type stained for PGL-1, which is at the two-cell stage. Wild-type and *mat-2(ax76) *embryos are oriented with anterior (maternal DNA, polar-body end) on the left. *chs-1(RNAi) *does not extrude polar bodies; therefore, it was not possible to determine the maternal end.

The *C. elegans *genome encodes two *chitin synthase (chs) *homologues. *chs-2 *is expressed in the pharynx and head neurons of larvae and adults, and *chs-1 *is expressed in the hermaphrodite germline, in eggs and in dauer larvae [56,57]. Consistent with its expression during early development, *chs-1 *RNAi blocked eggshell chitin production and resulted in a highly penetrant embryonic lethality that resembled *gna-2(qa705) *(Figure 6A,B). Embryos were fragile, osmotically sensitive and FM 4–64-permeable (Figure 6C,D). Meiotic timing was normal (Figure 3F), but embryos failed to extrude the polar bodies and had 12 sister chromatid pairs at meiosis II (100% of embryos; Table 1). Furthermore, stray chromatin detected at anaphase II (10% of embryos) suggested meiotic chromosome segregation defects (Table 1). Additionally, the SPCC failed to associate closely with the cortex (30% of embryos) and a pseudocleavage furrow was not detected (100% of embryos) (Table 1, Figure 4). PAR-3 and P granules were mislocalized and PIE-1 was undetectable (Figure 6E), showing that polarization was defective. Moreover, PAR-2::GFP was undetectable or mislocalized, and incubation in minimal EGM did not rescue localization (Figure 6E, Table 2). The nearly identical phenotypes of *gna-2(qa705) *and *chs-1(RNAi) *support the conclusion that chitin deficiency is responsible for the *gna-2(qa705) *Mel phenotype. The Pod phenotype of hypomorphic alleles of genes encoding APC subunits might suggest that APC activity is required for chitin synthesis. However, while *mat-2(ax76) *embryos at the non-permissive temperature (25°C) were arrested at meiotic metaphase I, they still produced a chitinous eggshell, showing that chitin synthesis does not depend on APC activity (Figure 6F). This result also indicates that the Pod phenotype of hypomorphic APC alleles does not result from chitin deficiency.

### *cej-1+B0280.5(coRNAi) *phenocopies *gna-2(qa705) *and *chs-1(RNAi)*

**Figure 7:**
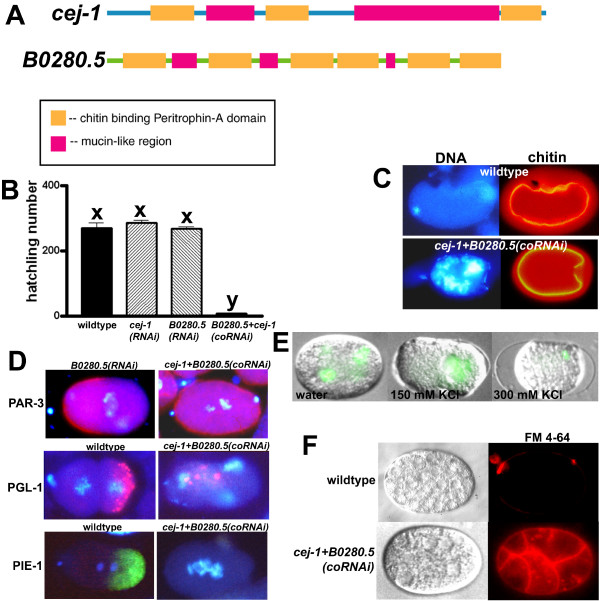
**Combined RNAi with the chitin-binding proteins, CEJ-1 and B0280.5, results in a *gna-2(qa705)*-like Pod phenotype**. (A) CEJ-1 and B0280.5 have multiple chitin binding Peritrophin-A domains and mucin-like regions. Amino terminus is on the left. (B) Hatchling number of wild type, *cej-1(RNAi)*, *B0280.5(RNAi) *or *cej-1+B0280.5(coRNAi) *(bars are mean ± SEM, *n *= 5, x,y: significantly different, *p *< 0.05). (C) DNA (Hoechst) and chitin staining of wild type or *cej-1*+*B0280.5(coRNAi)*. (D) Wild type or *B0280.5(RNAi) *(controls), or *cej-1*+*B0280.5(coRNAi) *stained for DNA (Hoechst; blue) and PAR-3 (red), PGL-1 (pink) or PIE-1 (green). Images are overlays of Hoechst and antibody staining. (E) Osmotic sensitivity of *cej-1+B0280.5(coRNAi) *embryos expressing histone H2B::GFP, dissected in water, 150 mM KCl or 300 mM KCl. Images are overlays of histone H2B::GFP (chromatin; green) and DIC. (F) FM 4–64 permeability of wild type or *cej-1+B0280.5*(coRNAi). DIC image is on the left in each pair. (C,D) Embryos are at the one-cell stage except wild type stained for PGL-1, which is at the two-cell stage. Wild-type and *B0280.5(RNAi) *embryos are oriented with anterior (maternal DNA, polar-body end) on the left. *cej-1+B0280.5(coRNAi) *does not extrude polar bodies; therefore, it was not possible to determine the maternal end.

*cej-1 *and *B0280.5 *encode proteins predicted to be secreted mucins with multiple chitin-binding Peritrophin-A domains (Figure 7A). Furthermore, *cej-1 *and *B0280.5 *are targets of the germline translational repressor GLD-1, and combined *cej-1 *and *B0280.5 *RNAi (*cej-1+B0280.5(coRNAi)*) has an embryonic lethal phenotype that resembles *gna-2(RNAi) *[22] (Figure 7B). These results suggested that CEJ-1 and B0280.5 might be required for eggshell assembly or function. *cej-1+B0280.5(coRNAi) *did not prevent eggshell chitin deposition (Figure 7C), and meiotic timing was normal (Figure 3F). However, embryos had stray chromatin at anaphase I (6% of embryos) and anaphase II (12% of embryos), failed to extrude the polar bodies (76% of embryos for polar-body 1, 82% of embryos for polar-body 2), and had 12 sister chromatid pairs at meiosis II (76% of embryos) (Table 1). Moreover, SPCC-cortical association failed (47% of embryos), and a pseudocleavage furrow was not detected (94% of embryos) (Table 1, Figure 4). Additionally, PAR-3 and P granules were mislocalized, PIE-1 was undetectable, PAR-2::GFP was undetectable or mislocalized, and PAR-2::GFP localization defects were not rescued by incubation in minimal EGM (Figure 7D; Table 2). Furthermore, like *gna-2(qa705) *or *chs-1(RNAi)*, *cej-1+B0280.5(coRNAi) *resulted in embryos that were osmotically sensitive and FM 4–64-permeable (Figure 7E,F). These findings support a model in which GNA-2 is required for CHS-1-dependent synthesis of eggshell chitin, which binds CEJ-1 and B0280.5, proteins with essential but overlapping roles in meiotic chromosome segregation, polar-body extrusion, osmotic barrier function and polarization.

### MBK-2 localization requires *chs-1*, but not *cej-1/B0280.5*

**Figure 8:**
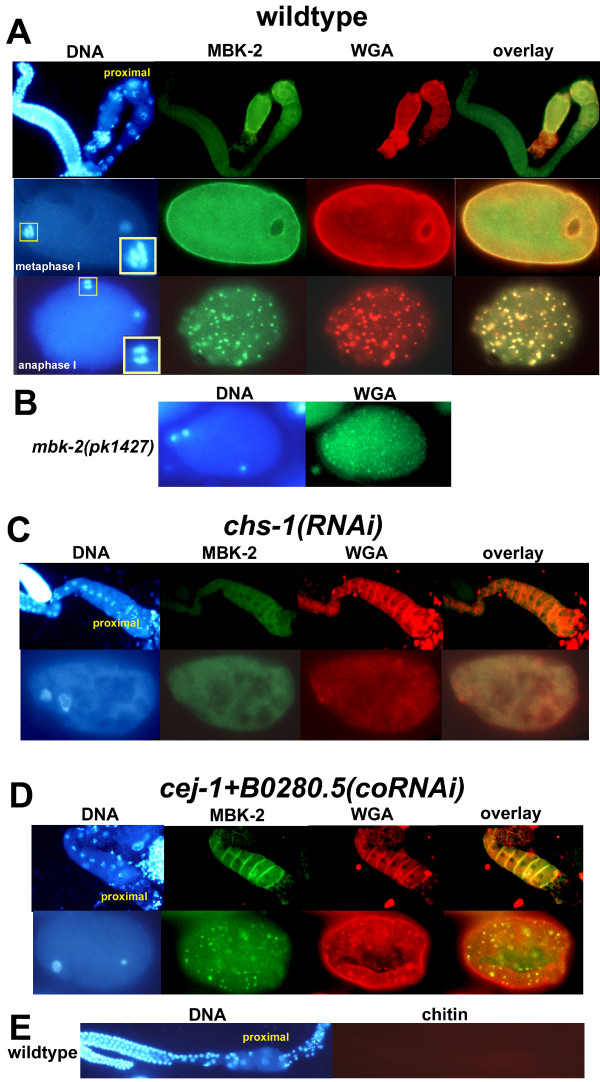
**MBK-2::GFP and WGA-G colocalize, and chs-1 is required for MBK-2 localization**. (A) Wild type expressing MBK-2::GFP, stained for DNA (Hoechst), GFP (MBK-2) or WGA-binding epitopes, in the germline (top row), or in embryos at meiotic metaphase I (middle row) or meiotic anaphase I (bottom row). In embryos, maternal DNA is shown enlarged in box on lower right hand of DNA images. (B) DNA (Hoechst) or WGA-binding epitopes in *mbk-2(pk1427) *(putative null). (C) *chs-1(RNAi) *expressing MBK-2::GFP and stained for DNA (Hoechst), GFP (MBK-2) or WGA-binding epitopes in the germline (top row), or in an early embryo. (D) *cej-1+B0280.5(coRNAi) *expressing MBK-2::GFP stained for DNA (Hoechst), GFP (MBK-2) or WGA-binding epitopes in the germline (top row), or in an early embryo. (E) DNA (Hoechst) or chitin in germline of wild type. (A-E) Proximal refers to the region of the germline near the spermatheca. Embryos are at the one-cell stage.

The DYRK-related kinase, MBK-2, is required for polarization and asymmetric division. Furthermore, like WGA-G (Figure 2G), it is cortically localized in oocytes in the proximal germline, and in the embryo its cortical distribution changes from uniform to dramatically punctate during meiosis [41]. To determine if MBK-2 and WGA-G colocalize, hermaphrodites expressing MBK-2::GFP were dissected, and germline and embryos were costained with a GFP antibody and WGA conjugated to tetramethylrhodamine-5-(and-6)-isothiocyanate (WGA-TRITC). WGA-G and MBK-2 colocalized in the cortex of oocytes in the proximal germline and in the cortex of newly fertilized embryos (Figure 8A). Furthermore, cortical WGA-G and MBK-2 punctae were completely coincident at anaphase of meiosis I (Figure 8A). MBK-2 localizes to centrosomes and chromosomes [41] by first mitosis, at which point, WGA-G punctae are no longer detected.

WGA-G and MBK-2 colocalize in the germline, and WGA-G was intact in *mbk-2(pk1427) *(Figure 8B), a putative null allele, suggesting that WGA-G could be required upstream of MBK-2 localization. In that case, disruption of embryonic WGA-G by *chs-1 *RNAi might also prevent MBK-2 localization. Hermaphrodites expressing MBK-2::GFP were subjected to *chs-1 *RNAi, and germline and embryos were costained with a GFP antibody and WGA-TRITC. Cortical MBK-2 was undetectable in oocytes and in embryos following *chs-1 *RNAi (Figure 8C). In contrast, neither MBK-2 nor WGA-G was mislocalized following *cej-1+B0280.5(coRNAi) *(Figure 8D). Chitin was not detected in oocytes in the germline, using the highly specific chitin-binding probe, CBD-F (Figure 8E), but *chs-1 *RNAi disrupted oocyte localization of MBK-2. The simplest explanation for these findings is that *chs-1 *RNA or CHS-1, rather than chitin itself, is required for MBK-2 localization. In this regard, chitin synthases [58] and the homologous vertebrate hyaluronan synthases [59], are unusual in their localization to the plasma membrane, rather than the endoplamic reticulum/Golgi apparatus. *Saccharomyces cerevesiae *chitin synthase-III is found in membranous organelles called chitosomes, and chitin synthase activation probably requires both enzyme phosphorylation and transport of chitosomes to the plasma membrane [58,60,61]. Therefore, one possibility is that MBK-2 may be localized to the oocyte cortex by virtue of its association with a CHS-1-containing compartment. Alternatively, chitin may be present in oocytes in the germline at a level that was too low to detect, or in a form that was masked by binding to a germline-expressed chitin-binding protein.

*chs-1 *RNAi disrupts MBK-2 localization, including the cortical patches that presage MBK-2-dependent APC degradation of MEI-1 and MEI-2. Furthermore, *chs-1(RNAi) *and *mbk-2(pk1427) *both fail to localize P granules [41], suggesting that MBK-2 deficiency might contribute to the *chs-1(RNAi) *phenotype. However, failed SPCC-cortical association in *chs-1(RNAi) *may be sufficient to explain P-granule mislocalization. Furthermore, *cej-1+B0280.5(coRNAi) *embryos mislocalize P granules, but MBK-2 localization is preserved. Taken together, these results suggest that MBK-2 mislocalization is not responsible for P-granule mislocalization in *chs-1(RNAi)*. Similarly, while *chs-1(RNAi) *and *mbk-2(pk1427) *both exhibit a multinucleated phenotype, *cej-1+B0280.5(coRNAi) *shares this phenotype; therefore, it is probably not attributable to MBK-2 mislocalization. The apparent insignificance of disruption of MBK-2 in *chs-1(RNAi) *may reflect the earlier and/or more severe developmental defects in *chs-1(RNAi)*.

## Discussion

### The eggshell is required for actin-dependent events at meiosis

Treatment of *C. elegans *embryos with latrunculin A, a drug that inhibits actin polymerization by sequestering G-actin, prevents polar-body extrusion and results in a phenotype of 12 sister chromatids at meiotic metaphase II [62]. Embryos deficient in PFN-1, an actin-binding protein, have a similar phenotype [62]. Here we demonstrate that in spite of normal meiotic timing, *gna-2(qa705)*, *chs-1(RNAi) *or *cej-1+B0280.5(coRNAi) *embryos have a latrunculin A-like meiotic phenotype, with failed polar-body extrusion, and 12 sister chromatid pairs at meiosis II. Therefore, a critical role of the eggshell in meiosis appears to be support of actin-dependent polar-body extrusion.

*C. elegans *males arise at a frequency of about 0.5%, by non-disjunction of the X chromosome, and a phenotype showing a high incidence of males (Him) is a common finding in mutants with meiotic chromosome-segregation defects [63]. *gna-2(qa705) *rescued by extrachromosomal array was Him, and stray chromatin was detected at meiosis I and meiosis II, in some *gna-2(qa705) *embryos. Furthermore, *chs-1(RNAi) *or *cej-1+B0280.5(coRNAi) *embryos also had stray chromatin at meiosis. These findings indicate that the eggshell is required for faithful meiotic chromosome segregation. Meiotic spindles localized to the embryonic cortex in *gna-2(qa705)*, *chs-1(RNAi) *or *cej-1+B0280.5(coRNAi) *embryos and meiosis occurred with normal timing, suggesting that gross meiotic spindle defects did not underlie the chromosome-segregation defects. However, it is possible that more subtle defects in spindle microtubule structure could explain the lagging chromosome phenotype. In starfish, and perhaps also in *Xenopus *and mouse [64-66], actin is essential for chromosome capture by the spindle during chromosome segregation. Therefore, the chromosome segregation defects identified in this study may also reflect a requirement for the eggshell in supporting actin-dependent chromosome capture.

FM 4–64 dye permeability is a reliable indicator of osmotic sensitivity in Pod mutants, and in wild-type embryos treated with a laser pulse to perforate the eggshell [5]. In wild-type embryos, the first polar body stained with FM 4–64, demonstrating that it is external to the barrier. Therefore, meiosis I and extrusion of the first polar body precede development of the osmotic/permeability barrier. Accordingly, the defects in meiosis I segregation and first polar-body extrusion in *gna-2(qa705)*, *chs-1(RNAi) *and *cej-1+B0280.5(coRNAi) *are not likely to be secondary to osmotic defects. CEJ-1 and B0280.5 bind chitin [11]; furthermore, they are substituted with chondroitin chains and are predicted to be mucins, which are highly hydrophilic and hydrating glycoproteins [11]. Chondroitin proteoglycans are required in the *C. elegans *hermaphrodite to prevent collapse of the developing vulva [12]. Furthermore, they are essential for polar-body extrusion and for the separation of the embryonic plasma membrane from the eggshell [12]. Therefore, analogous to the role of chondroitin-proteoglycans in the developing vulva, CEJ-1/B0280.5 may support actomyosin-mediated cytokinesis of polar bodies by generating a hydrated matrix.

### The eggshell is required for polarization, an actomyosin-dependent process

SPCC-cortical association failed in *gna-2(qa705)*, *chs-1(RNAi) *and *cej-1+B0280.5(coRNAi)*, and pseudocleavage furrow formation was not detected. Furthermore, in fixed embryos, cortical PAR-3 was not asymmetric, cortical PAR-2 was reduced and mislocalized, cytoplasmic P granules were not confined to the posterior, and cytoplasmic PIE-1 was undetectable. A perdurant meiotic spindle has been proposed as a possible explanation for reversal of polarization in *cul-2(RNAi) *[53]. However, in this study, we determined that meiotic spindle duration and meiotic timing were normal; therefore, the polarization defects associated with deficiency of eggshell chitin or CEJ-1/B0280.5 cannot be a consequence of an extended meiosis II and/or a perdurant meiosis II spindle.

The importance of actomyosin contraction in polarization is underscored by the previous observation that loss of function of actin-associated proteins, such as POD-1, NMY-2, MLC-4 or PFN-1, prevents both cytoplasmic flow and polarization [5,36,67,68]. Our results demonstrate that actomyosin-dependent polarization also requires eggshell chitin and CEJ-1/B0280.5. While most *gna-2(qa705), chs-1(RNAi) *and *cej-1+B0280.5(coRNAi) *embryos showed failed SPCC-cortical association, in a few embryos the SPCC appeared to associate closely with the cortex, but pseudocleavage was not seen. These results suggest that in addition to being required for SPCC movement to or association with the cortex, the eggshell may also be required subsequently for the anterior-directed cortical-domain movement that follows SPCC-cortical association.

### Pod mutants and the osmotic barrier

PAR mutants are polarity-defective, but osmotically insensitive. Therefore, polarization is not required for development of the osmotic barrier. In contrast, polarity is usually defective in osmotically sensitive mutants, and the Pod phenotype results from deficiency of a diverse group of proteins, including POD-1 (an actin-binding protein), APC subunits (MAT-1–3, EMB-27,30), enzymes required for fatty-acid synthesis (POD-2, an acetylCoA carboxylase, EMB-8, an NADPH-cytochrome P450 reductase, F32H2.5, a fatty acid synthase) [5,46,49], a ubiquitin C-terminal hydrolase required for actin-dependent processes (CYK-3) [54] and proteins required for eggshell synthesis (GNA-2, CHS-1, CEJ-1/B0280.5) (this study). These results indicate that polarization may require an osmotically appropriate environment, and that Pod-class polarity defects could be secondary to osmotic sensitivity. The results of our study, and those of Rappleye et al[49] for *emb-8(hc69)*, *emb-8(RNAi)*, *pod-2(RNAi) *or fatty-acid synthase (*F32H2.5*)(*RNAi*), showing that polarization was not rescued by incubation in isotonic medium, suggest that in some Pod mutants polarity defects may not simply be a consequence of osmotic defects. Intriguingly, Rappleye et al[49] also determined that *pod-2(ye60) *supplemented with long-chain fatty acids (C16-C22) were normally polarized when dissected in isotonic medium. Unfortunately, however, in that study it was not reported whether polarity was defective in embryos dissected in non-isotonic medium, probably reflecting the fact that osmotically sensitive embryos often burst (or crenate) without osmotic support. Therefore, while the results showed that a combination of lipid supplementation and osmotic support rescued polarization in *pod-2(ye60)*, it is not clear whether the rescue was provided by the lipids or by the isotonic medium. Additionally, the osmotically sensitive mutants *emb-8(t1462) *and 3-hydroxy-3-methylglutaryl coenzyme A (HMG-CoA) reductase (*F08F8.2*)(*RNAi*) were normally polarized when dissected in isotonic medium but no data were presented for embryos dissected in non-isotonic medium. As such, the question of whether or not polarization defects of Pod mutants are secondary to osmotic sensitivity remains unresolved.

As has been suggested for the ascaroside layer in *Ascaris *species [6], a role of the osmotic barrier in the *C. elegans *embryo may be to prevent desiccation after the egg is laid. The molecule(s) responsible for this barrier has not been identified. It may include lipid molecules of the inner eggshell layer playing a role analogous to that of ascarosides. However, EM images of *pod-1(ye11) *showed that all three eggshell layers appeared intact, but embryos were osmotically sensitive and dye-permeable [5]. Therefore, it seems likely that the *C. elegans *permeability barrier resides inside the inner eggshell layer, as suggested by our results showing that the first polar body (putatively in the inner eggshell layer) stains with FM 4–64, while the second does not. A deficiency of HMG-CoA reductase causes osmotic sensitivity, suggesting that the *C. elegans *osmotic barrier requires cholesterol [49]. Furthermore, as long-chain fatty acids [palmitate, stearate, oleate, linoleate, arachidonate, docosohenaenoate or phosphatidylinositol (linoleic + palmitoleic)] were insufficient to rescue the osmotic sensitivity of *pod-2(ye60) *[49], the barrier may require shorter-chain fatty acids or other malonyl CoA-derived molecules. In this study, we determined that *chs-1(RNAi) *or *cej-1+B0280.5(coRNAi) *embryos have defects in the osmotic/permeability barrier. These results suggest that the chitinous middle layer of the eggshell is required for synthesis/extrusion of the inner eggshell layer, which could be the osmotic barrier itself, or is required for subsequent synthesis/extrusion of molecule(s) comprising the osmotic barrier.

### The eggshell may provide a "skeleton" to support actomyosin contraction in the one-cell embryo

The eggshell is required for multiple early developmental events, including meiosis, polar-body extrusion, osmotic barrier function and polarization. However, while the eggshell is physically associated with the embryonic plasma membrane at meiosis I, by polarization, additional layers separate the chitinous eggshell from the embryonic plasma membrane. This indicates that the embryonic plasma membrane is in contact with different extracellular molecules at different stages in the development of the one-cell embryo, which may suggest that the eggshell serves distinct functions at different stages. For example, during meiosis I, when it is closely associated with the plasma membrane, the eggshell might act as a physical scaffold for cortical molecules overlying the meiotic spindle. Subsequently, during polar-body extrusion, the eggshell might provide a fluid space into which the polar bodies can be extruded. Additionally, from second polar-body extrusion onwards, the eggshell could be required primarily to provide an osmotic barrier. Moreover, as long-chain fatty-acid supplementation suggests that membrane properties are crucial for polarization [49], the eggshell might be required during polarization for integrity of the plasma membrane. While these or other distinct, separate roles could underlie the requirement for the eggshell in multiple early developmental processes, a common feature of these events may explain, at least in part, the requirement for the eggshell. Specifically, as meiosis, polar-body extrusion and polarization all appear to be actomyosin-dependent, it seems likely that the eggshell supports actomyosin-dependent contractile events.

At both the cellular and the whole-animal level, some form of skeletal support usually facilitates contractile events. For example, in motile mammalian cells, dynamic adhesion to the extracellular matrix supports the pulling forces of the actomyosin network that move the cell forward [70]. At the whole-animal level, skeletal-muscle contraction is facilitated by a skeleton, which can be internal or external, and rigid or hydrostatic. For example, in vertebrates and "hard-shelled" arthropods, contraction requires muscle attachment to the bony skeleton or to a rigid exoskeleton, respectively. In animals that lack a rigid skeleton, a hydrostatic skeleton can provide a zone of incompressible fluid to transmit the force of muscle contraction (reviewed by Chapman [71]). By way of example, coelomic fluid in annelids is propelled forward by a wave of contraction of circular and longitudinal muscles, which results in a net movement of the worm in the direction of coelomic fluid flow [71,72]. Furthermore, while crustaceans normally depend on a rigid exoskeleton for body muscle contraction, a hydrostatic skeleton is used during the post-molt, soft-shell stage [73]. Hydrostatic skeletons can also be utilized within the context of a rigid 'container'. For example, fluid in the gut of the nematode *Aphelenchoides *acts as a hydrostatic skeleton that facilitates movement either of gut contents or of the whole animal, depending on the coordination of body-wall muscle contraction. Specifically, when localized regions of dorsal and ventral body muscles contract in phase, gut contents are moved; conversely, a wave of alternating dorsal and ventral contraction results in sinusoidal locomotion of the worm.

CEJ-1 and B0280.5 bind chitin, and it is possible that they also bind, directly or indirectly, to plasma-membrane molecules that link to the actomyosin cytoskeleton. In this way, the eggshell and/or molecules in the PVF could act as a dynamic adhesive matrix to anchor focal actomyosin contraction during meiosis, polar-body extrusion and polarization, analogous to the role played by the extracellular matrix in supporting focal adhesion in migrating cells or extending axonal growth cones. Conversely, or additionally, as CEJ-1 and B0280.5 are chondroitin-modified and have multiple mucin domains, their role may reflect an ability to generate a fluid-filled space. In this case, transmission of actomyosin contractile forces in the one-cell *C. elegans *embryo may depend on the hydrated eggshell/PVF acting as a hydrostatic skeleton. This would be analogous to the situation in jumping spiders, where it has been proposed that a hydrostatic skeleton within a rigid exoskeleton allows application of a large amount of force to a small deformation, resulting in a forceful and quick movement [71]. In the case of polar-body extrusion, localized asymmetric force application may facilitate cytokinesis. Similarly, during the actomyosin-dependent ruffling stage that is coincident with movement of the large SPCC to the cortex, fluid translocated into the ingressing ruffles (and away from the non-ruffling adjacent membrane) might be required to generate sufficient force to propel the SPCC to the cortex.

## Conclusion

**Figure 9:**
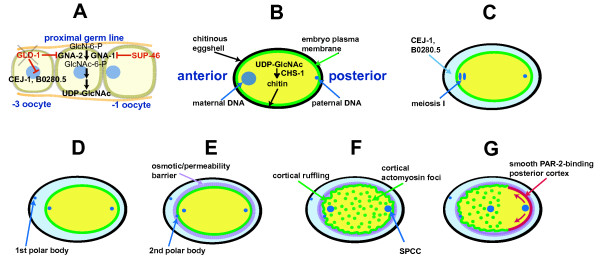
**The eggshell in development of the one-cell *C. elegans *embryo**. (A) *cej-1, B0280.5 *and *gna-2 *are released from GLD-1 translational repression in the proximal germline (indicated by 'x' superimposed on 'GLD-1'). GNA-2 supports synthesis of UDP-GlcNAc. GNA-1 expression is suppressed by SUP-46. '-1 oocyte' indicates the oocyte adjacent to the spermatheca. (B) CHS-1 synthesizes chitin (black) from UDP-GlcNAc. Chitin is deposited into the eggshell immediately after fertilization. (C) The chitin-binding proteins, CEJ-1 and/or B0280.5, bind chitin and water, resulting in the development of a hydrated matrix (pale blue), which is required for faithful chromosome segregation at meiosis I and for (D) extrusion of the first polar body. (E) Chitin and CEJ-1 and/or B0280.5 are also needed for extrusion of the osmotic/permeability barrier, for faithful meiosis II chromosome segregation and for extrusion of the second polar body. (F) The eggshell is required for association of the SPCC with the cortex, which (G) is associated with the initiation of polarization.

Figure 9 presents a summary of how early developmental events may be dependent on GNA-2, CHS-1 and the eggshell: (A) following relief of GLD-1-mediated *gna-2 *translational repression in oocytes in the proximal germline, GNA-2 is required for synthesis of UDP-GlcNAc for (B) eggshell chitin synthesis by CHS-1. (C) Chitin binds the chondroitin-modified chitin-binding proteins, CEJ-1 and/or B0280.5, made available following relief of GLD-1 translational repression. CEJ-1 and/or B0280.5 bind water, resulting in the development of a hydrated zone, which is required for faithful chromosome segregation at meiosis (I and II). (D) The hydrated zone is also required for actomyosin-dependent extrusion of the first polar body and, perhaps, for secretion of the inner layer of the eggshell. (E) After first polar-body extrusion, the eggshell is needed for exocytosis/secretion of the molecule(s) of the osmotic/permeability barrier. There is no direct evidence that secretion of the barrier is actomyosin-dependent. However, deficiency of the actin-binding protein, POD-1, results in a Pod phenotype combined with exocytosis/endocytosis defects [5], and our study shows that the timing of barrier extrusion is temporally coincident with that reported for redistribution of cortical actin into foci [69]. These findings are consistent with the possibility that osmotic/permeability barrier extrusion is actomyosin-dependent. The eggshell is also required for subsequent actomyosin-dependent extrusion of the second polar body. F) Following pronuclear decondensation, the eggshell is necessary for the SPCC to associate closely with the cortex, which is associated with the initiation of polarization. G) The eggshell may also be required during the ensuing phase of actomyosin-dependent development of asymmetric cortical PAR domains

## Methods

Standard methods were followed for molecular biology and nematode culture [63,74]. Unless otherwise stated, wild type refers to N2 worms fed OP50 bacteria.

### *gna-2(qa705) *isolation, rescue and suppressors

*gna-2(qa705) *was isolated by screening a chemical deletion library (1.1% ethyl methanesulphonate (EMS)-treated N2, generated in the laboratory of J. Culotti) by nested PCR using the primer pairs 5'-ACTCCGCCCCCAATCTTGTTGC-3' and 5'-AACCAGCGTACCGCACAGTTGC-3' and 5'-TTGTCGAAGGAAGGGGAGGAGC3' and 5'-AATCATTGTGGCGGCGAGGAGG-3'. Deletion breakpoints were established by sequencing the truncated PCR product. The *gna-2 *allele, *qa705*, was outcrossed 10 times to yield strain XA780 (*gna-2(qa705)/unc-109(n499)I*). The *gna-2(qa705)*-bearing chromosome was marked by recombination with *unc-55(e1170) *and balanced with *gld-1(q485) *to produce XA774 *(gld-1(q485)/gna-2(qa705) unc-55(e1170)I)*. Stable transformation rescue of *gna-2(qa705) *was demonstrated with a fragment of the cosmid T23G11(nucleotides 20340–23478, Genbank accession number Z81130) sub-cloned into pBSKS^- ^to yield the construct pWJ8. pAK42-1, a rescuing *gna-2::GFP *translational fusion, was generated by inserting the GFP coding sequence of pPD95.79 (a gift of A. Fire) into pWJ8 just prior to the stop codon of *gna-2 *(nucleotide 23146 of Genbank accession Z81130). Transgenic animals were generated by gonad injection [75]. Rol-6 (pRF4) was used as the visible co-injection marker, and empty pBSKS- was used to bulk out the arrays. Injections were conducted using a total DNA concentration of 150 ng/μl and a plasmid ratio of 1:9:5 rescuing construct: pBSKS^-^:pRF4. For pWJ8, wild-type hermaphrodites were injected, and arrays were passed into a *gna-2(qa705) unc-55(e1170*)*I *background. For pAK42-1, *gld-1(q485)/gna-2(qa705) unc-55(e1170) *hermaphrodites were injected, and rolling Uncs were selected. To confirm rescue, individual fertile, rolling Unc worms were genotyped for the deletion by PCR.

To identify suppressors of *gna-2(qa705) *an F2 screen was performed. *gld-1(q485)/gna-2(qa705) unc-55(e1170) *L4 hermaphrodites were mutagenized using EMS (50 mM). F2 progeny were fed *gna-2 *RNAi to enrich for suppressors, and Unc-55 F3s were picked and pooled (5/plate). Plates were examined for fertility and lines were established from hermaphrodites throwing only Unc progeny. A minimum of 6000 haploid genomes was screened. The recovered alleles were outcrossed 10 times, subjected to complementation analysis, and mapped to linkage group I. *qa707 *and *qa708 *were isolated from *gna-2(qa705) *and then reconstructed with *gna-2(qa705) *by recombination, with the aid of *unc-101(m1)*.

### Additional strains

The following additional strains were also constructed: XA781 (*gld-1(q485)/gna-2(qa705) unc55(e1170)I; unc-119(ed3) ruIs32 [unc-119(+)pie-1::GFP::H2B]III*), XA782 (*gld-1(q485)/gna-2(qa705) unc55(e1170)I; unc-119(ed3) III; ruIs57 [unc-119(+) pie-1::GFP::tubulin*]), XA785 (*gna-2(qa705) unc-55(e1170)I; qaEx747 [gna-2::GFP, pRF 4 pBS KS*^-^]), XA787 (*gna-2(qa705) unc-55(e1170)I; qaEx749 [gna-2::GFP pRF 4 pBS KS*^-^]), XA792 (*sup-46(qa707)I*), XA794 (*gna-2(qa705) unc55 (e1170) sup-46(qa707)I*), XA796 (*gna-2(qa705) unc55 (e1170) sup-46(qa708)I*), XA797 (*sup-46(qa708)I) and DE2 (dnEx2 [pRF 4 pBS KS*^-^]). We also obtained the following CGC strains: N2, CB4856, AZ212 (*unc-119(ed3); ruIs32 [unc-119(+) pie-1::GFP::H2B]III*), AZ244 (*unc-119(ed3) III; ruIs57 [unc-119(+) pie-1::GFP::tubulin*], DR1 (*unc-101(m1)I*), DR101 (*dpy-5(e61)unc-55(e1170) I*), DS97 (*mat-2(ax76)II*), JK1466(*gld-1(q485)/dpy-5(e61)unc-13(e51)I*), JH1576(*unc-119(ed3) III; axIs1140 [pie-1::gfp-mbk-2*]), JH1580(unc-24(e1172)mbk-2(pk1427)IV/nT1 [let-?(m435)](IV;V) and KK866 *(itIs153 [pie-1::gfp::par-2*, pRF4 N2 genomic DNA]).

### RNAi and hatchling number determination, and biochemical rescue

RNAi was performed by feeding [76]. The following RNAi feeding vectors were produced by cloning PCR-generated genomic sequence into the *Sma*1 site of pPD129.36 (a gift of A. Fire): pAK57-2 (*B0280.5 *primers 5'-TTCCTTCAAGGTGCGAGTTT-3' and 5'-AGTGCAAGCAGTGAATGACG-3'), pAK56-2 (*cej-1 *primers 5'-GAGACCACCACAGAAGCATATGCTCCAG-3' and 5'-GTGGTTTCAACGACTTCTGACTCGATC-3'), pAK55-1 (*chs-1 *primers 5'-TTCGTCCATCGGAAGTTTTC-3' and 5'CCAAGTACATGTCGATTGCG-3'). The *gna-2 *RNAi feeding vector, pAK38-2, was a PCR-generated cDNA fragment (primers 5'-CGGGATCCGATGCTATGAAAAAGGC-3' and 5'-GCTCTAGACTACAAGAGTGGCAGCACC-3') cloned into the *Bam*H1 and *Xba*I sites of pPD129.36. Double-stranded RNA production was induced in liquid culture, and bacteria were collected by centrifugation and spread on NGM plates supplemented with ampicillin, tetracycline and isopropyl-beta-D-thiogalactopyranoside (IPTG). *Escherichia coli *strain OP50 on unsupplemented NGM was used as a control, unless otherwise stated. For combined RNAi (co-RNAi), approximately equal amounts of each bacterial preparation were mixed before spreading on the plates.

To determine hatchling number, single L1/L2 larvae (P_o_) were transferred to individual plates and allowed to lay eggs. Total hatchling number/hermaphrodite was determined by aspirating F1 larvae prior to adulthood. For experiments quantifying % Him, the P_o _was transferred to a new plate each day until no new hatchlings were observed, and the sex of the hatchlings on each progeny plate was determined at the L4 or adult stage. Results were analysed by one-way analysis of variance (ANOVA) followed by Tukey's multiple comparison test. Hatchling number was determined, rather than brood size or % of brood that hatched, because brood-size measurements would have been inaccurate for the following reasons: (i) embryos with a defective eggshell are fragile and sometimes break as they are laid; these embryos would have be excluded from the brood count; (ii) embryos with a defective eggshell are often laid as part of a large cohesive mass, which precludes accurate quantification of the number of embryos in the mass; and (iii) *gna-2(qa705) *and *chs-1(RNAi) *embryos are pale brown and non-refractile, making them easily confused with (unfertilized) oocytes.

To test biochemical rescue of the *gna-2(qa705) *Mel phenotype, young adult Unc-55 hermaphrodites were picked from *gld-1(q485)/gna-2(qa705) unc55 (e1170)I *plates. UDP-GlcNAc was dissolved in water (10, 250, 100 or 250 mM) and injected into the rachis of a single gonad arm as described previously [75]. The number of hatchlings from each injected hermaphrodite was quantified by transferring individual hatchlings to single plates. Hermaphrodites throwing any wild-type or Gld-1 progeny were excluded from further analysis.

### HPLC

For HPLC analysis 1000 adult hermaphrodite wild type or *gna-2(qa705) *were collected in M9 buffer, washed three times in M9, incubated for 30 min at room temperature (RT), washed three times in H_2_O, resuspended in 50 μl H_2_O, frozen in dry ice/ethanol and stored at -80°C for no longer than 1 week. Samples were thawed rapidly, sonicated for 20 seconds, centrifuged, and the supernatant filtered and injected onto an anion exchange column (Zorbax SAX; Agilent Technologies, Chromatographic Specialities Inc., Brockville, ON). Detection was by ultraviolet absorption at 254 nm. UDP-GlcNAc, UDP GalNAc, UDP-Glc and UDP-Gal were quantified and UDP-GlcNAc and UDP-GalNAc were expressed as a percentage of UDP-Glc + UDP-Gal

### Microscopy

For fixed embryos, young adult hermaphrodites were washed in 150 mM KCl and transferred to 0.1% poly-lysine-coated slides. Embryos were released by cutting at the gonad, or gonads were released by cutting just behind the pharynx, followed by freeze-fracture [63]. Slides were fixed in 4°C MeOH for 10 min (PGL-1), in -20°C MeOH for 15 min (PAR-3) or in -20°C MeOH for 8 min (other antibodies, lectins and chitin-binding probe). After fixation, slides were washed for 2 min in phosphate-buffered slaine (PBS), and for 10 min in PBS with 0.5% Tween-20 (PBST), and were blocked in PBS containing 10% goat serum and 1% BSA (PBGS) for 30 min at RT. Primary antibodies were diluted in PBGS and applied overnight at 4°C at the following dilutions: anti-PGL-1 [77], 1:10000; anti-PIE-1, 1:100 [78]; anti-PAR-3 1:30 [79]. PAR-2::GFP and MBK-2::GFP (in fixed embryos), were detected by an anti-GFP antibody (1:100; Molecular Probes, Inc., Eugene OR). The following secondary antibodies (diluted in PBGS, applied at RT for 2–4 h) were used: goat anti-mouse (GAM)-fluorescein isothiocyanate (FITC) (1:100; Pierce, Rockford IL); goat anti-rabbit (GAR)-rhodamine (1:100; Jackson ImmunoResearch Laboratories, Inc, Westgrove PA); GAR-FITC (1:100; Sigma-Aldrich, Oakville ON). Lectins (LEA-FITC, SBA-FITC, STA-FITC, WGA-FITC, WGA-rhodamine; all Sigma-Aldrich) were diluted in PBGS (40 μg/ml) and applied overnight at 4°C. The chitin-binding probe (chitinase A1 chitin-binding domain fused to maltose binding protein and labeled with TRITC (CBD-F); gift of Y. Zhang, New England Biolabs, Inc., Ipswich MA) was diluted 1:250 in PBGS and applied overnight at 4°C. DNA was stained with Hoechst (Molecular Probes), then SlowFade (Molecular Probes) was added prior to mounting, and fluorescence was detected on a Leica Leitz DMRB microscope. To test osmotic sensitivity, living embryos were dissected in H_2_O, 150 or 300 mM KCl, transferred to the appropriate (H_2_O, 150 or 300 mM KCl) 2% agarose pad on a glass slide, and viewed by DIC. Compression by the glass coverslip was prevented by depositing embryos into the fluid-filled space that was created by removing a central disc from the pad. To test whether osmotic support could rescue PAR-2 polarization wild-type worms expressing PAR-2::GFP were fed *gna-2 *RNAi, *chs-1 *RNAi or *cej-1+B0280.5 *coRNAi, and embryos were dissected quickly in 150 mM KCl followed by freeze-fracture (control), or were dissected in minimal EGM [55] and incubated at 22°C for 30 min before freeze fracture. Instead of M9, 150 mM KCl was used as a control because *gna-2(RNAi)*, *chs-1(RNAi) *or *cej-1+B0280.5(coRNAi) *embryos dissected in M9 are very fragile and usually break, whereas the KCl provides some osmotic support. However, dissection and freezing was completed in <5 min, which should not allow for reversal of polarity defects.

To examine meiotic spindles, meiotic DNA segregation, polar-body extrusion, SPCC-cortical association, pseudocleavage furrow formation and pronuclear migration, wild-type or *gna-2(qa705) *hermaphrodites expressing tubulin::GFP or histone H2B::GFP were transferred to agarose pads, and embryos were examined *in utero*, using a Leica DMLSFA confocal microscope. The effect on meiotic DNA segregation, polar-body extrusion, SPCC-cortical association, pseudocleavage furrow formation and pronuclear migration of *chs-1(RNAi) *or *cej-1+B0280.5(coRNAi) *was also determined in worms expressing histone H2B::GFP. For meiosis duration (determined with tubulin::GFP), timing was defined as follows:

• Meiosis I: time from entry into the spermatheca until tubulin::GFP waned to a small spot (the tubulin::GFP signal does not wane to undetectable before the meiosis II spindle waxes). Time from entry into the spermatheca was used instead of nuclear envelope breakdown (NEBD) because it is easily and objectively assessed without DIC optics, and because it excludes any oocytes that undergo NEBD but fail to be ovulated (and fertilized) in a timely manner.

• Meiosis II: time from the end of meiosis I (defined above) until the meiosis II spindle waned to a very pale structure.

• Meiosis I + II: time from entry into the spermatheca until the meiosis II spindle waned to a very pale structure.

As an additional way to determine meiotic timing, we also examined chromosome segregation using histone H2B::GFP. Timing was defined as follows:

• Meiosis I: time from entry into the spermatheca until chromatin masses were separated (anaphase I).

• Meiosis II: time from entry into the spermatheca until chromatin masses were separated a second time (anaphase II).

• Pronuclear meeting: time from entry into the spermatheca until maternal and paternal pronuclei met.

Failure of the SPCC to associate closely with the cortex was scored as any embryo in which the sperm pronucleus (visualized with histone H2B::GFP) failed to come within half of the sperm pronuclear diameter of the cortex. Results were analysed by *t *test (tubulin::GFP experiment) or one-way ANOVA followed by Tukey's multiple comparison test (histone H2B::GFP experiment). Lipophilic dye permeability was tested by DIC/UV microscopy of living embryos dissected in 150 mM KCl containing 30 μM FM 4–64 (Molecular Probes).

## Abbreviations

GLD Germline differentiation abnormal

APC/C Anaphase-promoting complex/cyclosome

CBD Chitin-binding domain

CDC Cell division cycle related

CEJ Cell junction protein

CHS Chitin synthase

coA Coenzyme A

CUL Cullin

CYK Cytokinesis defect

DIC Differential interference contrast

DYRK Dual specificity tyrosine phosphorylation regulated kinase

EMB Embryogenesis abnormal

EMS Ethyl methanesulphonate

FITC Fluorescein isothiocyanate

GalNac N-acetyl-D-galactosamine

GAM Goat anti-mouse

GAR Goat anti-rabbit

GFP Green fluorescent protein

GlcNac N-acetylglucosamine

GLN Glutamine synthetase

GNA Glucosamine-6-phosphate N-acetyltransferase

GTPase Guanosine triphosphatase

LEA Lycopersicon esculentum agglutinin

MAPK Mitogen-activated protein kinase

MAT Metaphase to anaphase transition defect

MBK Minibrain kinase

MEI Meiosis defective

MEKK Mitogen-activated or extracellular signal-regulated protein kinase kinase

MEL Maternal effect lethal

MEX Muscle excess

MLC Myosin light chain

MSP Major sperm protein

NADPH Nicotinamide adenine dinucleotide phosphate (reduced form)

NMY Non-muscle myosin

OMA Oocyte maturation defective

PAR Embryonic partitioning abnormal

PDZ PSD-95/DLG/ZO-1

PFN Profilin

PIE Pharynx and intestine in excess

POD Polarity-osmotic defective

PVF Perivitelline fluid

SBA Soybean agglutinin

SPCC Sperm pronucleus/centrosome complex

SQV Squashed vulva

STA Solanum tuberosum agglutinin

TRITC Tetramethylrhodamine-5-(and-6)-isothiocyanate

UDP Uridine diphosphate

VAB Variable abnormal morphology

WGA Wheat germ agglutinin

ZIF Zinc finger-interacting protein

ZYG Zygote defective embryonic lethal

## Authors' contributions

WLJ isolated and characterized *gna-2(qa705)*, and performed microscopy and RNAi experiments. AK performed microinjections and conducted the suppressor screen. Plasmids and *C. elegans *strains were generated by WLJ and AK, and HPLC was conducted by JWD. WLJ, AK and JWD conceived of the study, and the manuscript was drafted by WLJ, with AK and JWD revising it critically. All authors read and approved the final manuscript.

## References

[B1] Kosinski M, McDonald K, Schwartz J, Yamamoto I, Greenstein D (2005). C. elegans sperm bud vesicles to deliver a meiotic maturation signal to distant oocytes. Development.

[B2] Yamamoto I, Kosinski ME, Greenstein D (2006). Start me up: Cell signaling and the journey from oocyte to embryo in C. elegans. Dev Dyn.

[B3] McNally KL, McNally FJ (2005). Fertilization initiates the transition from anaphase I to metaphase II during female meiosis in C. elegans. Dev Biol.

[B4] Wharton D (1980). Nematode egg-shells. Parasitology.

[B5] Rappleye CA, Paredez AR, Smith CW, McDonald KL, Aroian RV (1999). The coronin-like protein POD-1 is required for anterior-posterior axis formation and cellular architecture in the nematode Caenorhabditis elegans. Genes & Development.

[B6] Bird AF (1971). The structure of nematodes.

[B7] Perry RN (2002). The biology of Nematodes.

[B8] Foor WE (1967). Ultrastructural aspects of oocyte development and shell formation in Ascaris lumbricoides. J Parasitol.

[B9] Shen Z, Jacobs-Lorena M (1998). A type I peritrophic matrix protein from the malaria vector Anopheles gambiae binds to chitin. Cloning, expression, and characterization. J Biol Chem.

[B10] Tjoelker LW, Gosting L, Frey S, Hunter CL, Trong HL, Steiner B, Brammer H, Gray PW (2000). Structural and functional definition of the human chitinase chitin-binding domain. J Biol Chem.

[B11] Olson SK, Bishop JR, Yates JR, Oegema K, Esko JD (2006). Identification of novel chondroitin proteoglycans in Caenorhabditis elegans: embryonic cell division depends on CPG-1 and CPG-2. J Cell Biol.

[B12] Hwang HY, Horvitz HR (2002). The SQV-1 UDP-glucuronic acid decarboxylase and the SQV-7 nucleotide-sugar transporter may act in the Golgi apparatus to affect Caenorhabditis elegans vulval morphogenesis and embryonic development. Proc Natl Acad Sci USA.

[B13] Mei B, Kennedy MW, Beauchamp J, Komuniecki PR, Komuniecki R (1997). Secretion of a novel, developmentally regulated fatty acid-binding protein into the perivitelline fluid of the parasitic nematode, Ascaris suum. J Biol Chem.

[B14] Chrispeels M, Hindsgaul O, Paulson JC, Lowe J, Manzi A, Powell L, van Halbeek H (1999). Essentials of Glycobiology.

[B15] Chitwood BG, Chitwood MBH (1974). Introduction to nematology, Consolidated Edition.

[B16] Schierenberg E, Junkersdorf B (1992). The role of eggshell and underlying vitelline membrane for normal pattern formation in the early C. elegans embryo. Roux's Archives of Developmental Biology.

[B17] Piano F, Schetter AJ, Morton DG, Gunsalus KC, Reinke V, Kim SK, Kemphues KJ (2002). Gene clustering based on RNAi phenotypes of ovary-enriched genes in C. elegans. Curr Biol.

[B18] Kamath RS, Fraser AG, Dong Y, Poulin G, Durbin R, Gotta M, Kanapin A, LeBot N, Moreno S, Sohrmann M, Welchman DP, Zipperlen P, Ahringer J (2003). Systemic functional analysis of the Caenorhabditis elegans genome using RNAi. Nature.

[B19] Rual JF, Ceron J, Koreth J, Hao T, Nicot AS, Hirozane-Kishikawa T, Vandenhaute J, Orkin SH, Hill DE, van den Heuvel S, Vidal M (2004). Toward improving Caenorhabditis elegans phenome mapping with an ORFeome-based RNAi library. Genome Res.

[B20] Sonnichsen B, Koski LB, Walsh A, Marschall P, Neumann B, Brehm M, Alleaume AM, Artelt J, Bettencourt P, Cassin E, Hewitson M, Holz C, Khan M, Lazik S, Martin C, Nitzsche B, Ruer M, Stamford J, Winzi M, Heinkel R, Roder M, Finell J, Hantsch H, Jones SJ, Jones M, Piano F, Gunsalus KC, Oegema K, Gonczy P, Coulson A, Hyman AA, Echeverri CJ (2005). Full-genome RNAi profiling of early embryogenesis in Caenorhabditis elegans. Nature.

[B21] Jones AR, Schedl T (1995). Mutations in gld-1, a female germ cell-specific tumor suppressor gene in Caenorhabditis elegans, affect a conserved domain also found in Src-associated protein Sam68. Genes & Development.

[B22] Lee MH, Schedl T (2001). Identification of in vivo mRNA targets of GLD-1, a maxi-KH motif containing protein required for C. elegans germ cell development. Genes & Development.

[B23] Maddox AS, Habermann B, Desai A, Oegema K (2005). Distinct roles for two C. elegans anillins in the gonad and early embryo. Development.

[B24] Willis JH, Munro E, Lyczak R, Bowerman B (2006). Conditional dominant mutations in the Caenorhabditis elegans gene act-2 identify cytoplasmic and muscle roles for a redundant actin isoform. Mol Biol Cell.

[B25] Severson AF, Baillie DL, Bowerman B (2002). A Formin Homology protein and a profilin are required for cytokinesis and Arp2/3-independent assembly of cortical microfilaments in C. elegans. Curr Biol.

[B26] Munro E, Nance J, Priess JR (2004). Cortical flows powered by asymmetrical contraction transport PAR proteins to establish and maintain anterior-posterior polarity in the early C. elegans embryo. Dev Cell.

[B27] Cowan CR, Hyman AA (2004). Asymmetric cell division in C. elegans: cortical polarity and spindle positioning. Annu Rev Cell Dev Biol.

[B28] Wallenfang MR, Seydoux G (2000). Polarization of the anterior-posterior axis of C. elegans is a microtubule-directed process. Nature.

[B29] Cheeks RJ, Canman JC, Gabriel WN, Meyer N, Strome S, Goldstein B (2004). C. elegans PAR proteins function by mobilizing and stabilizing asymmetrically localized protein complexes. Curr Biol.

[B30] Nance J (2005). PAR proteins and the establishment of cell polarity during C. elegans development. Bioessays.

[B31] Cuenca AA, Schetter A, Aceto D, Kemphues K, Seydoux G (2003). Polarization of the C. elegans zygote proceeds via distinct establishment and maintenance phases. Development.

[B32] Kay AJ, Hunter CP (2001). CDC-42 regulates PAR protein localization and function to control cellular and embryonic polarity in C. elegans. Curr Biol.

[B33] Joberty G, Petersen C, Gao L, Macara IG (2000). The cell-polarity protein Par6 links Par3 and atypical protein kinase C to Cdc42. Nat Cell Biol.

[B34] Nishimura T, Yamaguchi T, Kato K, Yoshizawa M, Nabeshima Y, Ohno S, Hoshino M, Kaibuchi K (2005). PAR-6-PAR-3 mediates Cdc42-induced Rac activation through the Rac GEFs STEF/Tiam1. Nat Cell Biol.

[B35] Grill SW, Gonczy P, Stelzer EH, Hyman AA (2001). Polarity controls forces governing asymmetric spindle positioning in the Caenorhabditis elegans embryo. Nature.

[B36] Severson AF, Bowerman B (2003). Myosin and the PAR proteins polarize microfilament-dependent forces that shape and position mitotic spindles in Caenorhabditis elegans. J Cell Biol.

[B37] Ahringer J (2003). Control of cell polarity and mitotic spindle positioning in animal cells. Current Opinion in Cell Biology.

[B38] Lu C, Srayko M, Mains PE (2004). The Caenorhabditis elegans microtubule-severing complex MEI-1/MEI-2 katanin interacts differently with two superficially redundant beta-tubulin isotypes. Mol Biol Cell.

[B39] Dow MR, Mains PE (1998). Genetic and molecular characterization of the caenorhabditis elegans gene, mel-26, a postmeiotic negative regulator of mei-1, a meiotic-specific spindle component. Genetics.

[B40] Srayko M, Buster DW, Bazirgan OA, McNally FJ, Mains PE (2000). MEI-1/MEI-2 katanin-like microtubule severing activity is required for Caenorhabditis elegans meiosis. Genes & Development.

[B41] Pellettieri J, Reinke V, Kim SK, Seydoux G (2003). Coordinate activation of maternal protein degradation during the egg-to-embryo transition in C. elegans. Dev Cell.

[B42] Quintin S, Mains PE, Zinke A, Hyman AA (2003). The mbk-2 kinase is required for inactivation of MEI-1/katanin in the one-cell Caenorhabditis elegans embryo. EMBO Rep.

[B43] Pang KM, Ishidate T, Nakamura K, Shirayama M, Trzepacz C, Schubert CM, Priess JR, Mello CC (2004). The minibrain kinase homolog, mbk-2, is required for spindle positioning and asymmetric cell division in early C. elegans embryos. Dev Biol.

[B44] Pintard L, Willis JH, Willems A, Johnson JL, Srayko M, Kurz T, Glaser S, Mains PE, Tyers M, Bowerman B, Peter M (2003). The BTB protein MEL-26 is a substrate-specific adaptor of the CUL-3 ubiquitin-ligase. Nature.

[B45] Shirayama M, Soto MC, Ishidate T, Kim S, Nakamura K, Bei Y, van den Heuvel S, Mello CC (2006). The Conserved Kinases CDK-1, GSK-3, KIN-19, and MBK-2 Promote OMA-1 Destruction to Regulate the Oocyte-to-Embryo Transition in C. elegans. Curr Biol.

[B46] Rappleye CA, Tagawa A, Lyczak R, Bowerman B, Aroian RV (2002). The anaphase-promoting complex and separin are required for embryonic anterior-posterior axis formation. Dev Cell.

[B47] Shakes DC, Sadler PL, Schumacher JM, Abdolrasulnia M, Golden A (2003). Developmental defects observed in hypomorphic anaphase-promoting complex mutants are linked to cell cycle abnormalities. Development.

[B48] Tagawa A, Rappleye CA, Aroian RV (2001). pod-2, along with pod-1, defines a new class of genes required for polarity in the early Caenorhabditis elegans embryo. Dev Biol.

[B49] Rappleye CA, Tagawa A, Le Bot N, Ahringer J, Aroian RV (2003). Involvement of fatty acid pathways and cortical interaction of the pronuclear complex in Caenorhabditis elegans embryonic polarity. BMC Dev Biol.

[B50] Gotta M, Ahringer J (2001). Axis determination in C. elegans: initiating and transducing polarity. Current opinion in genetics & development.

[B51] Natsuka S, Kawaguchi M, Wada Y, Ichikawa A, Ikura K, Hase S (2005). Characterization of wheat germ agglutinin ligand on soluble glycoproteins in Caenorhabditis elegans. J Biochem (Tokyo).

[B52] Sonneville R, Gonczy P (2004). Zyg-11 and cul-2 regulate progression through meiosis II and polarity establishment in C. elegans. Development.

[B53] Liu J, Vasudevan S, Kipreos ET (2004). CUL-2 and ZYG-11 promote meiotic anaphase II and the proper placement of the anterior-posterior axis in C. elegans. Development.

[B54] Kaitna S, Schnabel H, Schnabel R, Hyman AA, Glotzer M (2002). A ubiquitin C-terminal hydrolase is required to maintain osmotic balance and execute actin-dependent processes in the early C. elegans embryo. J Cell Sci.

[B55] Edgar LG (1995). Blastomere culture and analysis. Methods Cell Biol.

[B56] Reinke V, Gil IS, Ward S, Kazmer K (2004). Genome-wide germline-enriched and sex-biased expression profiles in Caenorhabditis elegans. Development.

[B57] Zhang Y, Foster JM, Nelson LS, Ma D, Carlow CK (2005). The chitin synthase genes chs-1 and chs-2 are essential for C. elegans development and responsible for chitin deposition in the eggshell and pharynx, respectively. Dev Biol.

[B58] Cohen E (2001). Chitin synthesis and inhibition: a revisit. Pest Manag Sci.

[B59] Lee JY, Spicer AP (2000). Hyaluronan: a multifunctional, megaDalton, stealth molecule. Curr Opin Cell Biol.

[B60] Kozubowski L, Panek H, Rosenthal A, Bloecher A, DeMarini DJ, Tatchell K (2003). A Bni4-Glc7 phosphatase complex that recruits chitin synthase to the site of bud emergence. Mol Biol Cell.

[B61] Valdivia RH, Schekman R (2003). The yeasts Rho1p and Pkc1p regulate the transport of chitin synthase III (Chs3p) from internal stores to the plasma membrane. Proc Natl Acad Sci USA.

[B62] Yang HY, McNally K, McNally FJ (2003). MEI-1/katanin is required for translocation of the meiosis I spindle to the oocyte cortex in C elegans. Dev Biol.

[B63] Sulston J, Hodgkin J (1988). The Nematode Caenorhabditis elegans.

[B64] Lenart P, Bacher CP, Daigle N, Hand AR, Eils R, Terasaki M, Ellenberg J (2005). A contractile nuclear actin network drives chromosome congression in oocytes. Nature.

[B65] Gard DL, Cha BJ, Roeder AD (1995). F-actin is required for spindle anchoring and rotation in Xenopus oocytes: a re-examination of the effects of cytochalasin B on oocyte maturation. Zygote.

[B66] Leader B, Lim H, Carabatsos MJ, Harrington A, Ecsedy J, Pellman D, Maas R, Leder P (2002). Formin-2, polyploidy, hypofertility and positioning of the meiotic spindle in mouse oocytes. Nat Cell Biol.

[B67] Guo S, Kemphues KJ (1996). A non-muscle myosin required for embryonic polarity in Caenorhabditis elegans. Nature.

[B68] Shelton CA, Carter JC, Ellis GC, Bowerman B (1999). The nonmuscle myosin regulatory light chain gene mlc-4 is required for cytokinesis, anterior-posterior polarity, and body morphology during Caenorhabditis elegans embryogenesis. J Cell Biol.

[B69] Strome S (1986). Fluorescence visualization of the distribution of microfilaments in gonads and early embryos of the nematode Caenorhabditis elegans. J Cell Biol.

[B70] Small JV, Kaverina I (2003). Microtubules meet substrate adhesions to arrange cell polarity. Curr Opin Cell Biol.

[B71] Chapman G (1975). Versitility of hydraulic systems. J Exp Zool.

[B72] Quillin KJ (2000). Ontogenetic scaling of burrowing forces in the earthworm Lumbricus terrestris. J Exp Biol.

[B73] Taylor JR, Kier WM (2003). Switching skeletons: hydrostatic support in molting crabs. Science.

[B74] Sambrook J, Fritsch EF, Maniatis T (1989). Molecular cloning. A laboratory manual.

[B75] Mello CC, Kramer JM, Stinchcomb D, Ambros V (1991). Efficient gene transfer in C. elegans: extrachromosomal maintenance and integration of transforming sequences. Embo J.

[B76] Timmons L, Court DL, Fire A (2001). Ingestion of bacterially expressed dsRNAs can produce specific and potent genetic interference in Caenorhabditis elegans. Gene.

[B77] Kawasaki I, Shim YH, Kirchner J, Kaminker J, Wood WB, Strome S (1998). PGL-1, a predicted RNA-binding component of germ granules, is essential for fertility in C. elegans. Cell.

[B78] Tenenhaus C, Schubert C, Seydoux G (1998). Genetic requirements for PIE-1 localization and inhibition of gene expression in the embryonic germ lineage of Caenorhabditis elegans. Dev Biol.

[B79] Etemad-Moghadam B, Guo S, Kemphues KJ (1995). Asymmetrically distributed PAR-3 protein contributes to cell polarity and spindle alignment in early C. elegans embryos. Cell.

